# Integrating Agriculture and Health Research for Development: LCIRAH as an Interdisciplinary Programme to Address a Global Challenge

**DOI:** 10.1002/gch2.201700104

**Published:** 2018-04-03

**Authors:** Jeff Waage, Laura Cornelsen, Alan D. Dangour, Rosemary Green, Barbara Häsler, Elizabeth Hull, Deborah Johnston, Suneetha Kadiyala, Karen Lock, Bhavani Shankar, Richard D. Smith, Helen L. Walls

**Affiliations:** ^1^ London School of Hygiene and Tropical Medicine Keppel Street London WC1E 7HT UK; ^2^ Royal Veterinary College 4 Royal College Street London NW1 0TU UK; ^3^ School of Oriental and African Studies Thornhaugh Street London WC1H 0XG UK

**Keywords:** agriculture, development, environment, health, interdisciplinary research, nutrition

## Abstract

The multiple burdens of persistent undernutrition and micronutrient deficiencies, along with the rapidly growing rates of overweight, obesity, and associated chronic diseases, are major challenges globally. The role of agriculture and the food system in meeting these challenges is very poorly understood. Achieving food security and addressing malnutrition in all its forms, a Sustainable Development Goal, requires an understanding of how changing food systems affect health outcomes and the development of new tools to design and evaluate interventions. An interinstitutional programme to address this interdisciplinary research challenge is described. Over the past seven years, the Leverhulme Centre for Integrative Research on Agriculture and Health has built a portfolio of successful and innovative research, trained a new cadre of interdisciplinary researchers in “Agri‐Health,” and built an international research community with a particular focus on strengthening research capacity in low‐ and middle‐income countries. The evolution of this programme is described, and key factors contributing to its success are discussed that may be of general value in designing interdisciplinary research programmes directed at supporting global development goals.

## 1 Introduction

Today's food systems are failing to deliver adequate and appropriate nutrition.^1^ Undernutrition, resulting from insufficient and inadequate diets and repeated and chronic infections affects millions of households, particularly in low‐ and middle‐income countries. In the same countries burdened by high rates of undernutrition there has also been a rapid growth in diets composed of inexpensive refined flours, fats, salt, and sugar, contributing to an epidemic of overweight, obesity, and associated diseases such as diabetes and cardiovascular disease. Across both rural and urban communities, many diets are also lacking essential micronutrients for health and development.

Historically, action in the health sector to reduce undernutrition has involved “nutrition‐specific” interventions such as education to encourage breast feeding and food supplements for infants. In 2013, the Maternal and Child Nutrition Series in the Lancet^2^ estimated that a set of these actions, if fully implemented in regions with undernutrition, would reduce undernutrition by not more than 25%. In recent years, to address the remaining burden of undernutrition, attention has therefore turned to other forms of intervention, such as “nutrition‐sensitive” actions to improve livelihoods and changes in agriculture and food systems to improve diets and food safety. Agriculture and food systems have also come under increasing scrutiny as causes of food‐borne diseases, particularly in low‐ and middle‐income countries.^3^

Many low‐ and middle‐income countries have seen dramatic improvement in the production of agricultural staples over recent decades, particularly cereals, driven by policies directed at national economic growth and food security. Many in the agriculture sector assumed that producing more staple foods would mean less hunger and improved nutrition and health. However, the evidence for such improvement arising from agricultural development is limited—even for agricultural interventions intended to improve nutrition.^4^, ^5^

This lack of evidence has generated intense interest today in understanding the processes by which agricultural and food system change affects the composition, quality, and safety of diets, and how this can in turn improve nutrition and reduce disease associated with poor diets and unsafe food.

International development has been slow to see the link between agriculture, food, nutrition, and health. The Millennium Development Goals recognized neither the improvement of agriculture nor nutrition as key development objectives, or as a contributor to obtaining the improved health and education goals on which they were principally focused. Decades of isolation between agriculture and health development communities had generated little interest and no methods to evaluate the effects on health of agricultural investments. Research on the interactions between agriculture, food, nutrition, and health, and the tools to evaluate these and design and test better interventions, were largely lacking.

However, in recent years, high‐level international interest in agriculture, nutrition, and health has grown. In 2013, a Nutrition for Growth Event hosted by the UK Government's Department for International Development (DFID) and the Children's Investment Fund Foundation was held in London, where donors and governments pledged over $4bn to support nutrition interventions including new and substantial investment in agriculture and food systems for nutrition. This event led to the establishment in 2013 of the Global Panel on Agriculture and Food Systems for Nutrition (https://www.glopan.org/), to advocate the development of nutrition sensitive policies in the agricultural sector. In 2014, FAO and WHO held the Second International Conference on Nutrition which identified specific actions across agriculture and other sectors to address malnutrition. The Sustainable Development Goals (SDGs), adopted in 2015, included the specific goal to “end hunger, achieve food security, improved nutrition, and promote sustainable agriculture.” Agriculture, nutrition, and health are now clearly linked development objectives in the SDGs, and the new challenge is how sectoral investments in each can be governed and implemented so as to generate positive outcomes.^6^

With this new development focus on linking agriculture and health outcomes, pressure has intensified on the scientific community to identify “what works?” to improve agriculture's contribution to nutrition and health. This has led to new efforts to create an intersectoral and interdisciplinary approach that brings together agricultural and health development research communities and their different disciplinary approaches and tools.

In this paper, we describe an initiative to integrate agricultural and health research communities for improved nutrition and health, the Leverhulme Centre for Integrative Research on Agriculture and Health (LCIRAH, www.lcirah.ac.uk/). Starting in 2011, LCIRAH has grown from an academic idea into a project funded by the Leverhulme Trust, and then into a diverse programme of integrated research projects, contributing along the way to creating a new field of research, and a new community of researchers that is contributing to this development challenge. We focus here on how LCIRAH was developed, the challenges we faced and the lessons we have learned that may be of general interest to interdisciplinary research programmes directed at global development. Note that we shall refer to LCIRAH as an *interdisciplinary* programme, for convenience, to describe work which involves research integration across both sectors (e.g., agriculture, health, environment) and their component disciplines (e.g., biology, economics, sociology).

## 2 Establishment of LCIRAH

LCIRAH has its origins in a prior collaboration focused on interdisciplinary research for development: the London International Development Centre (LIDC, www.lidc.org.uk). LIDC was established in 2008 as a formal collaboration between six specialist Colleges of the University of London, and coordinated a programme of intercollegiate and interdisciplinary discussions to identify interest and opportunities for development research collaboration. Over the past decade, LIDC has generated a range of cross‐College, interdisciplinary collaborations in development research in areas such as impact evaluation, education, and health, and assessment of the Millennium Development Goals and subsequent Sustainable Development Goals. Through LIDC, researchers from several Colleges discovered a common interest in collaborating across agriculture and health research. These included the School of Oriental and African Studies, with its programmes in international agricultural development, anthropology, economics, and food studies; the London School of Hygiene & Tropical Medicine, with its focus on public health and nutrition research in developing countries; the Royal Veterinary College, with its interest to link agriculture and health through livestock systems research and work on food safety, public health, and “One Health” approaches;^7^ and the London School of Pharmacy, with its programme on pharmacognosy and the use of foods as medicines in many cultures. Senior researchers from these Colleges agreed to meet regularly to explore opportunities for collaboration.

Commitment to academic collaboration was encouraged by the prospect of funding, and this new LIDC working group set their sights on a broad Leverhulme Trust call on “Embedding of Emerging Disciplines” that invited proposals to develop new disciplines needed to address 21st century problems. We submitted a proposal in 2009 entitled “Building an Agri‐Health Discipline to Link Agricultural and Health Research,” and created a concept, “Agri‐Health” as a unifying approach and methodology for understanding the relationships between agricultural production and population health, and the factors that drive them both.

**Figure 1:**
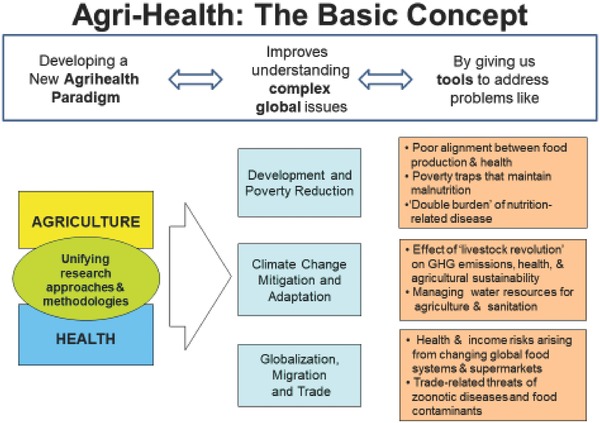
The original Agri‐Health concept as proposed to Leverhulme Trust in 2009.

While today the interaction between health and agriculture is well articulated and appreciated, in 2009 there was far less understanding of both the problem and its potential solutions. In our proposal, we therefore identified some key global development issues that we felt would benefit from an integrated approach, and presented these as a graphical argument shown in **Figure** **1**. We proposed that decoupled agriculture and health systems and policies were preventing effective action to address these challenges. In responding to the “new discipline” focus of the Leverhulme Trust call, we argued that historical isolation of health and agriculture across governmental, intergovernmental, and private sector systems could, to a large extent, be traced to the sectoral and disciplinary isolation of research and training in universities. Hence change had to start there, breaking down the research silos between these sectors and disciplines.

Our application was successful and we began LCIRAH in 2010 with a budget of £3.5m over five years. The reader may surmise correctly that our title, the Leverhulme Centre for Integrative Research on Agriculture and Health, was created by a committee, perhaps our first success, along with Figure 1, in finding a common language with which we could work together across sectors and disciplines.

In 2010, before the LCIRAH programme formally started, we organized an international conference entitled “Building an Integrated Agriculture and Health Agenda: Issues for International Research and Policy” that attracted over 150 international researchers across the disciplinary spectrum. Ideas from this meeting were captured and used in designing LCIRAH's programme, starting a continuing practice of using meetings and workshops to constantly revisit and reshape the LCIRAH collaboration.

## 3 Building an Interdisciplinary Team and Programme

Leverhulme Trust support was strongly focused on funding researchers; new academic staff, Postdoctoral researchers and PhDs, but did not extend to research project costs. We asked for two five‐year professorial appointments in different Colleges to provide leadership, and for a number of Ph.D., postdoctoral, and three‐year lecturer positions. We distributed these positions across our colleges, to embed an Agri‐Health research capacity in each.

Our sponsor, the Leverhulme Trust, was very flexible, allowing us to propose that “our research programme will not be planned in advance, as exploring new, interdisciplinary collaboration demands a strong degree of open‐endedness.” We established a regular cross‐College Management Committee (MC) comprising ten natural and social scientists from agriculture and health sectors. The MC had the role of developing our programme, agreeing research projects within it, and finding new funding to support this. The MC was supported by an MC member acting as chair, and a (usually) full‐time manager, who together comprised an LCIRAH Secretariat with a brief to support MC activities, administer the Leverhulme Trust grant, and help with new project development.

To plan our initial Ph.D. and postdoctoral appointments, the MC brainstormed five thematic areas: poverty and development; diet, globalization, and food quality; sustainability, environment, and climate change; agriculture, health, and food‐borne and zoonotic diseases; and metrics for agriculture and health. Workshops were held to develop research ideas for each of these areas, and it was agreed that all research work carried out by Ph.D.'s and postdoctoral researchers would be interdisciplinary with supervision from at least two researchers in different sectors and disciplines, usually in different colleges. The first round of studentships and postdoctoral positions was made to teams of MC members in 2010. Subsequently, Ph.D. studentships were opened to applications from interdisciplinary teams in all LCIRAH member colleges.

As LCIRAH developed, it made a concerted and repeated effort to gather ideas on Agri‐Health from experts in different fields, and to discuss and evolve its research programme. We organized workshops in areas related to Agri‐Health where there was little interdisciplinary thinking, such as climate change effects, farmers' occupational health, and zoonotic and food‐borne diseases. We also organized away‐days for developing our programme in 2010, 2012, and 2015. At our 2012 away‐day we iterated and agreed a common overarching research question for our interdisciplinary work: *How do we achieve sustainable food and agriculture systems which promote health and well‐being for all people?*

In 2011, we established an Advisory Group of leading researchers interested in agriculture and health from different sectors and disciplines. They met with us in 2012 and 2014, and then less formally as expert advisors on specific topics, and provided valuable external perspectives that caused us to modify our course on more than one occasion.

Monthly MC meetings created a regular opportunity to share new funding opportunities and to decide whether and how LCIRAH MC members would develop new joint research proposals for these. LCIRAH was very successful at attracting grants from a growing number of funding agency calls for interdisciplinary research, possibly because we could build more convincing proposals than those from more ad hoc interdisciplinary partnerships created specifically to respond to new calls.

## 4 Expanding the LCIRAH Research Platform

As a result of these efforts by the MC, the Leverhulme Trust‐funded project soon became a platform for a portfolio of new research projects funded by a range of sponsors. LCIRAH became the “glue” that linked these projects, adding value to each of them and using them to generate even more research opportunities.

Most of our new grants were significantly more restricted than the LCIRAH grant, with very specific activities and outputs expected by the sponsoring bodies. This made developing a portfolio of complementary and interactive projects challenging. The MC played a key role in shaping the growth of this research portfolio, prioritizing specific areas for new proposals to create balance across our research themes, ensuring that new projects continued to be interdisciplinary and cross‐institutional, and distributing new grant leadership across colleges.

**Figure 2:**
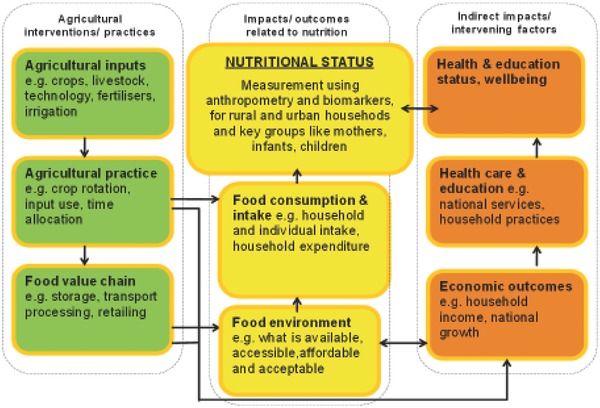
LCIRAH's conceptual framework for identifying research gaps, adapted from Turner et al.^8^

A crucial grant for LCIRAH was awarded by DFID in 2011 to map current and planned research on agriculture for improved nutrition and to analyze key research gaps.^8^ This was a modest scoping exercise, but it allowed LCIRAH to develop, with new partners, a conceptual framework for Agri‐Health research and to identify key research needs. This framework is illustrated in **Figure** **2**. Each block is an area of research, and linking these to existing publications revealed gaps in research studies that took agricultural interventions (green) through to nutritional outcomes (yellow), and a general gap in research on indirect effects of agricultural income (orange) on improving nutrition by improving health and education. The study revealed that little research on agriculture interventions aimed at improving nutrition ever measured effects on the food environment, food consumption, or nutritional status, and that the link between agricultural innovation, economic outcomes, and consequent health effects was profoundly under‐researched.

DFID then took the findings from this study to develop a programme of investment in Agri‐Health research, the competitive tender for which was won by LCIRAH. This new project, Innovative Methods and Metrics for Agriculture Nutrition Action (IMMANA, immana.lcirah.ac.uk/), included a programme of competitive research grants and fellowships, and support for a new international effort to build an Agri‐Health research community, which is described in Section 6. IMMANA helped LCIRAH to develop an international, coordinating role in Agri‐Health research, building relationships with donors and researchers worldwide, and to establish an important partnership with Tufts University's Friedman School of Nutrition, and its extensive network of researchers operating under the USAID‐funded Nutrition Innovation Lab. IMMANA grants, awarded internationally, have generated innovative methodologies for Agri‐Health, while fellowships have given outstanding postdoctoral researchers working in low‐ and middle‐income countries the opportunity to learn skills from Agri‐Health groups, building important institutional partnerships for the future.

DFID‐funded projects have been a strong feature of LCIRAH development, including not only those just mentioned but also support to LCIRAH participation in the programme, Leveraging Agriculture for Nutrition in South Asia (LANSA), and a support role to the Global Panel on Agriculture and Food Systems for Nutrition, to be discussed in Section 7. More recently, LCIRAH has won new agriculture‐nutrition‐related research projects in Asia and Africa, funded by joint calls from DFID and the Bill and Melinda Gates Foundation (BMGF).

In 2015, the Wellcome Trust, a leading funder of basic health research, started a new interdisciplinary initiative on environmental drivers of health outcomes, entitled Our Planet Our Health. LCIRAH won three competitive research projects from this initiative, all relating to novel research on food systems, environment, and health, followed by a major, five‐year programme, Sustainable and Healthy Food Systems (SHEFS) which started in 2017. LCIRAH's body of work around sustainable and healthy food systems has been an area of particular research innovation, and we highlight some of its outputs in Box 1..

Box 1. **LCIRAH research on environmental dimensions of Agri‐Health challenges**

Agriculture and the wider food system is a major contributor to environmental impacts. The production of food generates up to 30% of global greenhouse gas emissions and accounts for substantial proportions of land‐use change and global water consumption, situating population‐level dietary choices as a key driver of future planetary health.^9^ This critical nexus between food systems, health, and the environment has necessarily become a key question for LCIRAH in recent years. Our early work estimated the greenhouse gas emissions of UK diets and modeled the health effects of dietary shifts toward low emission, healthy diets.^10^, ^11^ More recently, we have for the first time estimated the greenhouse gas^12^ and water footprints^13^ of distinct dietary patterns in India and estimated the potential of dietary shifts to reduce vulnerability of the agricultural system to future predicted water scarcity.^14^ Recently, LCIRAH has focused on palm oil as a commodity that exemplifies the complex tradeoffs between economic, environmental, and health outcomes in modern food systems. Palm oil confers significant economic benefits as a cheap and versatile food ingredient, but oil palm production has been linked to deforestation and increased greenhouse gas emissions, while palm oil's high saturated fat content has been associated with adverse cardiovascular health outcomes. Working with researcher partners in Thailand, LCIRAH has developed an innovative simulation modeling framework that links a whole‐economy model of Thailand, including its food sector, with models of cardiovascular health outcomes, land‐use change and greenhouse gas emissions. This quantitative and interdisciplinary simulation framework is being complemented with qualitative stakeholder analysis to examine key policy questions,^15^ such as palm oil taxation, and strategic development of palm oil for biofuel‐use, rather than food‐use.

While much LCIRAH research addresses the effects of agriculture and food systems on nutritional outcomes, there has also been a research focus on how agricultural change affects other aspects of health, particularly infectious disease. In a partnership with the Agriculture for Nutrition and Health programme (A4NH) of the Consultative Group on International Agricultural Research (CGIAR) and the International Livestock Research Institute (ILRI), from 2017 LCIRAH has initiated a programme of research on the role of changing agricultural landscapes and vector‐borne diseases like malaria, on emerging zoonotic diseases associated with livestock systems, on food safety, and on the interactions of animal and human health practices in generating antimicrobial resistance in low‐ and middle‐income countries.

Bringing the different nutrition‐ and disease‐related aspects of LCIRAH's Agri‐Health research together has revealed complex trade‐offs and particular challenges for interdisciplinary research. This is a relatively unexplored area and Box 2. describes LCIRAH research that has begun to explore it.

Box 2. **LCIRAH research on nutritional and health trade‐offs in food systems**

While both health and nutrition research communities focus on the well‐being of people, the two research fields have evolved in parallel, generating different paradigms, approaches, methods, tools and protocols.^16^ These differences make interdisciplinary work on agriculture, nutrition, and health complex and challenging. An LCIRAH team has tackled this challenge in a programme on animal sourced food (ASF) chains led by Liverpool University and based at the International Livestock Research Institute in Nairobi. They have investigated the relationships between ASF production, distribution, consumption, nutrition outcomes, and health in poor urban communities. ASFs, including meat, eggs, and dairy, provide a range of essential nutrients of high quality and bioavailability, but can also carry food safety and noncommunicable diseases risks that require careful management if ASF is promoted for improving nutrition. Researchers from a range of disciplines characterized the structure, flows, processes, deficiencies, and potential health or nutrition risks of key livestock chains supplying Nairobi.^17^, ^18^, ^19^ These were linked to studies on the nutritional status of nonpregnant women of reproductive age and 1 to 3‐year‐old children^20^ to assess how this ASF supply could address nutrient gaps. Other studies analyzed the demand for ASF consumption, including drivers and barriers.^21^ All streams of research combine to provide a rich picture of the role of ASFs in urban populations and their potential impact in terms of nutrition and health. This is providing a basis for future work to design nutritious and safe food environments for the urban poor.

**Figure 3:**
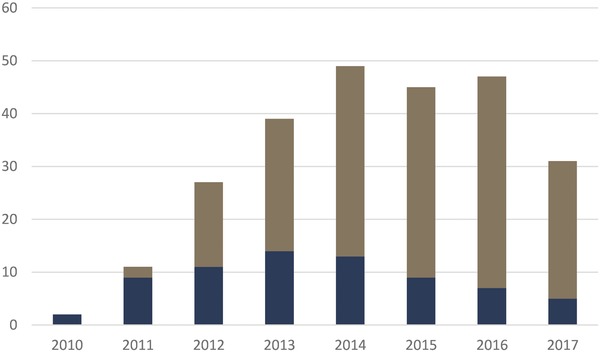
The growth of LCIRAH's research project portfolio, presented as numbers of projects operating in each year (e.g., a three‐year project started in 2011 would be counted in 2011, 2012, and 2013). LCIRAH's initial Leverhulme Trust funded projects are shown in blue, and subsequent projects from other sources in brown.

**Figure** **3** shows the growth in LCIRAH projects over time. The original LCIRAH‐funded Ph.D. and postdoctoral projects were followed by a growing number of new, externally funded projects that included some additional Ph.D.'s but mostly research grants, ranging greatly in size from less than £100k to over £7m.

These included several major and ongoing research grants described above, including IMMANA (£7.2m from 2014), LANSA (£7.5m from 2013), DFID‐BMGF nutrition projects (£3.1m from 2016), Wellcome Trust OPOH grants (£1.1m from 2014), SHEFS (£5m from 2017), and Improving Human Health (£3.2 from 2017). In terms of total grant value, the Leverhulme Trust's initial investment of £3.5m generated by the end of 2017 over £20m in Agri‐Health research projects led by the LCIRAH team. Using funds remaining from Leverhulme Trust support and contributions from other projects, LCIRAH plans from 2018 to maintain its modest, coordinating secretariat activity while seeking new core funding on the basis of its success to date.

## 5 Developing a Next Generation of Agri‐Health Researchers

LCIRAH built its initial research programme around Ph.D. studentships and postdoctoral fellowships shared by scientists from different disciplines and institutions. This not only served LCIRAH's aim of generating interdisciplinary research, but also it created the challenge to develop a next generation of Agri‐Health researchers.

A shared workspace was provided to Ph.D. and postdoctoral researchers from different colleges and projects to encourage interdisciplinary interactions and team building. They became known as the “Junior Team,” and in 2011, monthly MC meetings were extended to have a follow‐on Team Meetings for the MC, Junior Team, and visiting fellows, at which we discussed Agri‐Health research areas and shared presentations from staff and students on their work. As LCIRAH progressed, we were able to add additional Ph.D.'s to the Junior Team with funding from other sources. From 2010 to 2017, 22 Ph.D.'s have been trained through LCIRAH, including the 12 original positions funded by Leverhulme Trust.

One of LCIRAH's original objectives was to establish, halfway through the grant, an interdisciplinary master's degree in Agri‐Health. However, our early experience suggested that the Agri‐Health area was so new that students trained at the M.Sc. level would find limited employment opportunities in institutions that were still strongly disciplinary. We chose instead to develop focused short course training to reach disciplinary specialists and give them new interdisciplinary skills. A project funded by Irish Aid allowed LCIRAH to develop an open‐access online training course in agriculture, nutrition, and health which has been widely accessed by nutritionists, agriculturalists, and other professionals (https://tinyurl.com/yahaa2jb). LCIRAH also helped to build a cross‐University consortium, Innovative Food Systems Teaching and Learning (IFSTAL, https://www.ifstal.ac.uk), led by Oxford University, to which we added extramural Agri‐Health training to ongoing M.Sc. and Ph.D. programmes in seven higher education institutions, which now reaches over 1000 students.

## 6 Building a New Interdisciplinary Research Community

From the outset, LCIRAH had a stated objective to “build a new disciplinary area.” This could not be done by simply conducting new research and training in a few London Colleges. It required a broader, international dimension. The stimulus to start this international LCIRAH activity came when, in February 2011, the International Food Policy Research Institute (IFPRI), a CGIAR center, organized a major international conference entitled Leveraging Agriculture for Improving Nutrition and Health in New Delhi, India.^22^ At the IFPRI event, it became clear that an opportunity existed to help link a scattered collection of research activities on measuring impacts of agriculture on nutrition and health in different countries. Mostly donor‐funded and university‐based, it was felt that linking up these activities could facilitate the exchange and further development of research methods.

LCIRAH volunteered at the IFPRI conference to help develop this research cooperation and in May 2011 convened a meeting, Measuring Effects of Agri‐Health Interventions, focused on sharing and discussing methodological approaches. Following this, LCIRAH created a cross‐institutional working group on Agri‐Health methods, involving 11 institutions in Europe, USA, Africa, and Latin America. This group and its associated Agri‐Health Newsletter was short‐lived, but the idea of regular meetings to share research survived and became LCIRAH's annual international research conference on Agri‐Health, held in London.

Subsequent annual conferences covered a range of themes, with an emphasis on sharing new methods and approaches to research. Papers for presentation were selected competitively by the LCIRAH MC and Junior Team on the basis of their originality and quality, and a large poster session ensured that most participants had a chance to present their work. Despite growing numbers, LCIRAH made a commitment to have all sessions in plenary, in order to ensure disciplinary specialists learned from papers that covered the entire spectrum of Agri‐Health research.

At the 2013 LCIRAH conference, a vision for an even larger global research engagement was developed. LCIRAH and the CGIAR's A4NH, with which LCIRAH had partnered since the 2011 IFPRI Conference, organized a small workshop for research leaders from northern and southern institution to discuss creation of an agriculture, nutrition, and health “academy,” with a mission to share innovative research in Agri‐Health, stimulate new research, strengthen the capacity of the research community to undertake Agri‐Health research, and facilitate the uptake of research evidence in policies and programming. The plan was to expand the annual LCIRAH conference into an annual research and learning event, and relocate it in the developing world in order to better build Agri‐Health research capacity there. The IMMANA grant, awarded in 2014, gave LCIRAH resources to join with A4NH in establishing the first Agriculture Nutrition and Health (ANH) Academy (anh‐academy.org) meeting, a week‐long event in 2016 in Addis Ababa. This attracted over 300 researchers, with a strong African representation. The week comprised 2 d of interdisciplinary research training and mentoring sessions, volunteered by leading researchers from around the world, followed by a 3 d international scientific conference with papers and posters selected competitively. A second Academy week followed in Kathmandu in 2017, in partnership with Nepalese partners and the USAID‐funded Nutrition Innovation Lab, attracting larger numbers and strong Asian participation. Besides scientists, these meetings have drawn in research sponsors and policy advisors, creating opportunities for this growing research community to influence its own development and delivery.

## 7 Delivering Development Impact

As a development‐facing activity, LCIRAH aimed to contribute ultimately to improving the lives and well‐being of people, particularly the poor and disadvantaged. A simple “impact pathway” for LCIRAH would see the new research methods and their activities used to generate research and evidence to inform new policy interventions that contribute to improved well‐being. A programme like LCIRAH, therefore, could contribute toward impact by producing research methods and results as *outputs*, and by helping others to use these outputs to generate evidence‐informed policy *outcomes*.

LCIRAH's most direct contribution to research outputs and outcomes has been through the projects that its researchers have carried out themselves, their publications, and how these have been used. Since 2010 LCIRAH members, its MC and Junior Team, have generated 130 publications in peer‐reviewed journals across agriculture and health disciplines. LCIRAH research projects would often use their outputs as opportunities to engage policy makers. For instance, LCIRAH and A4NH undertook a systematic review on agriculture, women's time use, and nutrition in low‐ and middle‐income countries^23^ and used its finding to encourage the UK All‐Party Parliamentary Group on Agriculture and Food for Development to hold a meeting in 2015 entitled “It is Time: Valuing Women's Time in Nutrition Research and Policy,” attended by Members of Parliament, donors NGOs, and development specialists.

A particular impact that interdisciplinary research programmes like LCIRAH can have is to influence the trajectory of research funding, by helping donors to identify research gaps and priorities. The LCIRAH study illustrated in Figure 2, which led to the IMMANA project and influenced other funding initiatives, is a case in point.

LCIRAH conferences and ANH Academy meetings have created many opportunities for national policy makers and representatives from international organizations to engage with researchers about evidence to shape better interventions. This took the form of keynotes and panel discussions, from which both policy makers and researchers benefited.

**Figure 4:**
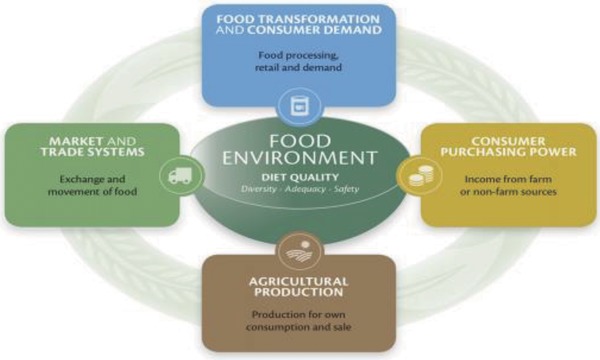
Conceptual framework developed by the Global Panel to help engage policy makers.^25^ This framework shows how policies in different domains, for example, promotion of biofortified crops and food safety regulation, integrate to affect the food environment for consumers and hence their diets and health.

LCIRAH's recognition as a platform for research in Agri‐Health also led to its more direct involvement in policy support. Following the establishment of the Global Panel on Agriculture and Food Systems for Nutrition in 2013, its sponsors, DFID, and BMGF, asked LIDC to host the Global Panel Secretariat so that it could draw on LCIRAH technical support. Global Panel members needed simple, evidence‐based messages for their work with policy makers. The Secretariat engaged the LCIRAH Junior Team to analyze the literature on agriculture–nutrition interactions in order to create a simple conceptual framework to help policy makers understand how their actions contribute to nutritional outcomes.^24^ This was then used by LCIRAH researchers and partners to generate a schematic, which showed how actions in different policy domains influence food systems, diets, and nutrition.^25^ These domains are shown in **Figure** **4**. For each policy domain, an analysis was made of interventions and the strength of scientific evidence for their effectiveness. Interventions with a strong evidence base became the subject of short Technical and Policy Briefs for Panel members to use with the relevant government ministries. For instance, a brief on biofortification was prepared for the agricultural policy sector, on managing food price volatility for the marketing and trade sector, and on food safety interventions for policy makers working in the area of food transformation and consumer demand. LCIRAH also helped to convene research experts for the Global Panel foresight study “Food systems and diets: Facing the challenges of the 21st century”^26^ which has been influential in shaping policy dialogue in this area.

## 8 LCIRAH as an Interdisciplinary Research Programme to Address a Global Challenge

In the remainder of this paper, we examine several aspects of the LCIRAH experience that may be generally relevant to developing impactful interdisciplinary research programmes to address global challenges. We relate these “lessons learned” to three key elements of LCIRAH's programme described above: building an interdisciplinary research programme, training a next generation of researchers, and creating a global research community. In **Table** **1**, we present a simple, informal summary of lessons learned against these programme elements, and explore each in more detail below.

**Table 1:** Summary of key “lessons learned” in the LCIRAH programme of potential broader relevance to interdisciplinary research programmes to address global challenges

LCIRAH programme element	Key lessons learned
Building an interdisciplinary research programme	Meet frequently to manage collaborative work and develop new projects Provide external resources to facilitate involvement in interdisciplinary work Exploit the integrative power of conceptual frameworks Focus on research to develop new methods and metrics
Training a next generation of interdisciplinary researchers	Ensure co‐supervision by different disciplinary experts Build interdisciplinary skills on a strong disciplinary skill base Nurture the innovative contributions of young researchers
Building a global interdisciplinary research community	Locate annual research meetings in developing countries Provide learning labs for researchers to build their interdisciplinary skills Adopt an inclusive partnership approach

### 8.1 Building an Interdisciplinary Research Initiative

LCIRAH's success is directly related to the commitment of its very diverse group of scientists to interdisciplinary collaboration. A monthly gathering ensured that the MC would meet frequently to manage collaborative work and develop new projects. The agreed requirement that all new work involve explicit supervision from two or more disciplinary leads reinforced these interactions, as did the early group effort to draft a common overarching question for our research. At MC meetings, collective decisions were made about how to invest research funds and what new proposals to develop. Regular workshops to explore new areas ensured that LCIRAH kept at the cutting edge of Agri‐Health thinking and could develop pilot projects in new areas, such as those described in Boxes Box 1. and Box 2..

However, LCIRAH began as an interdisciplinary collaboration of essentially disciplinary scientists with careers in strongly disciplinary departments and institutions. To encourage interested disciplinary scientists to devote a significant proportion of their time to more complex projects involving other disciplines, LCIRAH needed to provide external resources to facilitate involvement in interdisciplinary work. These resources were modest but critical. They involved the work of LCIRAH's very small secretariat, assisted by MC members, in organizing regular meetings and workshops, finding research partners from other disciplines and institutions, identify interdisciplinary funding opportunities, and assisting researchers with grant proposals to unfamiliar funding bodies.

Perhaps the most substantial resource that scientists used to support and develop new interdisciplinary collaborations was the LCIRAH Ph.D. studentships and postdoctoral fellowships. In this way, the focus of Leverhulme Trust support to fund new people rather than new projects was fortuitous. Ph.D. and postdoctoral funds created a unique opportunity for specialist researchers to explore interdisciplinary collaboration in a gradual manner without enormous personal time investment. The challenges faced by Ph.D.'s and postdoctoral researchers working under diverse disciplinary supervision will be discussed later, but from the LCIRAH researchers' perspective, these opportunities have been a key feature in starting and maintaining interdisciplinary collaboration.

Another important interdisciplinary learning from the LCIRAH programme has been to exploit the integrative power of conceptual frameworks. With their background in international development, many LCIRAH researchers were already familiar with development concepts of impact pathways and theories of change that relate interventions, often technical, to outcomes arising from them, and ultimately to impact on the well‐being of populations.^27^ LCIRAH used concepts like these to bring together different disciplines to cocreate an understanding of how agricultural and food system interventions led to different nutritional and health outcomes. The framework in Figure 2, from the pivotal LCIRAH study that led to the IMMANA programme, is a good example of using conceptual frameworks to understand the relationship between agricultural and food system interventions and health outcomes that revealed the gaps in methodologies and evidence that helped to focus LCIRAH's programme. Similar integrating frameworks also helped LCIRAH researchers to frame LCIRAH's original plan (Figure 1) and to later engage policy makers (Figure 4). Other conceptual frameworks in Agri‐Health^28^ have had a similar impact in engaging disciplinary scientists around interdisciplinary ideas.

Finally, we believe a significant feature of LCIRAH's success as an interdisciplinary initiative has been its decision to focus on research to develop new integrative methods and metrics, rather than on research to design or evaluate specific interventions and their uptake. LCIRAH research has been more theoretical than practical, in that it has focused on developing methods to measure and understand the nutrition and health outcomes of agricultural and food system changes and interventions. This has kept LCIRAH a strongly scientific exercise, rather than a development one, which appealed to its academic participants and to the international scientific community which it has facilitated. In this way, LCIRAH has challenged interdisciplinary research at its most sensitive point—where different disciplines have different, often incompatible, approaches to measuring key relationships.

This scientific research focus meant that LCIRAH encountered the well‐known problem of finding a home for an interdisciplinary research output in a world where most international journals are disciplinary. However, we found that having an MC comprising researchers with strong disciplinary experience helped us to draft papers attractive to disciplinary journals, while the innovative nature of interdisciplinary approaches and findings also helped us to publish in some very high‐impact journals.

### 8.2 Training a New Generation of Interdisciplinary Researchers

Ph.D. studentships provided the initial mechanisms for building LCIRAH's programme. From the outset, LCIRAH envisaged postgraduate training as a mechanism to build a new cadre of researchers with the skills to respond to future challenges across Agri‐Health. It also gave disciplinary specialists a relatively easy way to explore interdisciplinary ideas.

LCIRAH's commitment to ensure co‐supervision by different disciplinary experts was successful in that it led to many innovative research projects and publications. However, it was not long before LCIRAH Ph.D.'s were concerned about the risk of turning out “jacks of all trades and masters of none.” They found the task of becoming “interdisciplinarians,” proficient in more than one discipline, quite challenging, particularly as their highly disciplinary supervisors often had no experience of interdisciplinary approaches themselves. This stimulated much discussion in the LCIRAH Team and ultimately the learning that, at least in the early stages of a programme, we should build interdisciplinary skills on a strong disciplinary skill‐base. For Ph.D.'s, this meant that training should involve both developing strong skills in a core discipline as well as additional interdisciplinary skills to understand and work with other disciplines. This concept was also extended to the external training programmes that we developed, involving on‐line training in Agri‐Health for researchers from any discipline, and the IFSTAL programme that augmented disciplinary degrees with specific interdisciplinary training.

LCIRAH's most important lesson from its training activities was to encourage the innovative contributions of young researchers. Students and postdoctoral fellows were at the cutting edge of LCIRAH's new interdisciplinary approach and showed considerable imagination in addressing its challenges. It was LCIRAH's Junior Team that organized workshops on interdisciplinarity, inviting experts in this field to advise LCIRAH and sharing conclusions and key publications^29^, ^30^ with their supervisors and others. To build their own familiarity with different disciplines and methods, the Ph.D.'s and postdoctoral researchers developed peer‐to‐peer learning activities. These included “Agri‐Health 101,” a lecture series that team members delivered to each other on the “need‐to‐know” basics of each of their disciplines, covering areas including agricultural economics, nutrition, and qualitative research methods. Finally, the Junior Team organized a highly interdisciplinary collaborative project, outside their main research, to learn to work together as a team. While Ph.D. and project priorities prevented its completion, the project was highly valued as a skill‐ and team‐building exercise.

Involvement of the Junior Team in developing LCIRAH benefited both the programme and their own research careers, helping them to build research contacts, soft skills, and publications. The Junior Team took on key roles in organizing the annual LCIRAH conferences, and subsequently ANH Academy weeks, where their Agri‐Health 101 was one of the most popular learning labs. They also undertook to author, with the support of the journal Food Security, a peer‐reviewed summary paper following every LCIRAH and Academy conference.^31^, ^32^, ^33^, ^34^

### 8.3 Building a Global Interdisciplinary Research Community

When LCIRAH began, Agri‐Health‐relevant research was fragmented and scattered across isolated projects, each developing approaches independently. As described above, LCIRAH saw an opportunity to bring this research community together to share experiences in order to accelerate the development of better research methods. We quickly learned that this argument was attractive to donor organizations that were funding these separate groups and projects.

**Figure 5:**
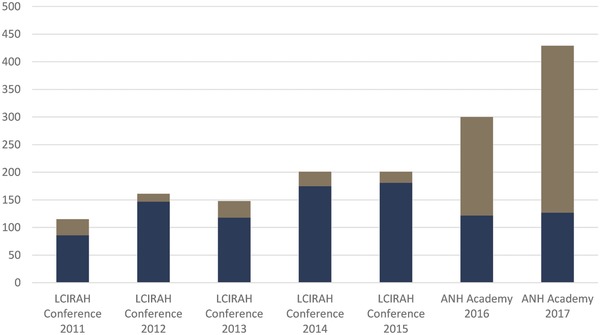
Numbers of participants attending LCIRAH international research conferences and ANH Academy weeks. Participants from DAC countries are shown in brown, and non‐DAC countries in blue. DAC countries are low‐ and middle‐income countries designated by the Organization for Economic Cooperation and Development (OECD) as eligible for international development assistance (http://www.oecd.org/dac/stats/daclist.htm).

As described earlier, LCIRAH's annual research conference was its principle strategy for global engagement. **Figure** **5** shows the number of participants at successive LCIRAH conferences and, more recently, ANH Academy weeks into which they were incorporated. The growth in participation at LCIRAH conferences between 2011 and 2015 was encouraging. But we found that London‐based conferences were not attracting researchers from low‐ and middle‐income countries (DAC countries in the figure), even though the majority of studies presented in papers and posters at the conferences were sited in those countries. The researchers making these presentations came largely from institutions in Europe, North America, Australia, and other high‐income countries.

With the establishment of the Academy we decided to locate annual research meetings in developing countries and encourage global participation. This gamble was successful and provided an important lesson about how to internationalize an interdisciplinary research community. We decided to relocate the conference to LMICs, moving it annually between Africa and Asia. Figure 5 shows that Academy meetings in Africa and Asia have attracted far more participants from LMICs than those held in Europe.

Surveys of ANH Academy Week participants from LMICs revealed that they were particularly attracted by our creation of learning labs for researchers to build their interdisciplinary skills. These proved particularly attractive to researchers from LMICS. At the same time, continuing a high profile international scientific conference maintained involvement of top research groups from high‐income countries. To further encourage their continued participation, we invited leading experts from these groups in high‐income countries to design and present learning labs ANH Academy weeks. They responded enthusiastically, bringing innovative and interactive learning sessions from their own universities or overseas training programmes.

Finally, we learned that adopting an inclusive partnership approach to the ANH Academy and its meetings rapidly attracted new partners and interest. Started by LCIRAH and A4NH, the ANH Academy sought from the outset the active involvement of other local and international institutions and groups. Other research centers, university groups and donor‐funded programmes quickly joined and contributed learning labs and events within and around the conference programme. They appreciated the inclusion of their logos on ANH Academy branding and their membership of committees to design its scientific programme. As the ANH Academy became an annual “go‐to” meeting place for this area of research, donors helped to further enlarge this community by asking that their grant holders attend ANH Academy Week, and by using the ANH Academy website as the platform for advertising new funding opportunities in Agri‐Health.

## 9 Conclusions

LCIRAH has achieved considerable progress in operationalizing an interdisciplinary research collaboration to address the global challenge of “achieving sustainable food and agriculture systems which promote health and well‐being for all people.” This has involved a focus on facilitation and skill‐building for researchers from a range of disciplines across agriculture, nutrition, health, and environmental sectors. This paper has described the diversity of ways in which this team‐building has been done. A critical driver of our progress has also been imaginative and flexible funding, which we used to make it easier for researchers to participate in new interdisciplinary work from their disciplinary base, letting them explore new ideas without expected outputs. On this investment, provided for LCIRAH by the Leverhulme Trust, a large portfolio of more focused research has been built. Another critical driver was institutional vision, in our case one shared by several specialist Colleges of the University of London. It is significant that these colleges, seven years on, have taken eight LCIRAH‐funded appointments and postdoctoral researchers onto their full‐time academic staff and built Agri‐Health research, and continuing interinstitutional collaboration, into their institutional strategies.

LCIRAH research teams have generated an evidence base on interactions between agriculture and health that will guide development investment agendas, as well as methods to support design and evaluation of interventions to deliver these agendas. Along the way, we realized the opportunity to achieve even greater impact than our research projects could generate on their own. This was to help create a global research community in this new area and a next generation of skilled interdisciplinary researchers. We have described the range of postgraduate training activities and the construction of a global learning initiative that have contributed to this. Given the novelty of many interdisciplinary initiatives to address global challenges, this dual strategy of investing in your own programme of research while also investing strongly in building interdisciplinary research capacity internationally may be the best way to contribute to solving complex development challenges.

## Conflict of Interest

The authors declare no conflict of interest.
